# Cadmium-induced annulus fibrosus cell senescence contributes to intervertebral disc degeneration via the JNK/p53 signaling pathway

**DOI:** 10.22038/IJBMS.2024.72312.15728

**Published:** 2024

**Authors:** Xin Liu, Wenjie Zhao, Man Hu, Yu Zhang, Jingcheng Wang, Liang Zhang

**Affiliations:** 1 Department of Orthopedics, Clinical Medical College of Yangzhou University, Yangzhou 225001, Jiangsu, China; 2 Department of Orthopedics, Dalian Medical University, Dalian 116000, Liaoning, China; # These authors contributed equally to this work

**Keywords:** Annulus fibrosus, Cadmium, c-Jun N-terminal kinase, Intervertebral disc-degeneration, Senescence, Tumor suppressor protein- p53

## Abstract

**Objective(s):**

Investigating the impact of cadmium (Cd) on annulus fibrosus (AF) cells and its potential mechanism was the purpose of the current study.

**Materials and Methods:**

Cd was cultivated in different concentrations (0, 1, 5, 10, and 20 μM) on AF cells and the potential effects of the metal were assessed. Using the CCK-8 method, cell viability and proliferation were identified. Using transcriptome analysis, the annulus fibrosus cells were sequenced both with and without cadmium chloride. The EdU method was used to determine the rate of cell proliferation; senescence-associated β-galactosidase (SA-β-Gal) staining was used to determine the number of positive cells; and western blot, RT-PCR, and immunofluorescence were used to determine the protein and mRNA expression of senescence-associated proteins (p16, p21, and p53) and c-Jun N-terminal kinase (JNK).

**Results:**

According to the findings, Cd has the ability to increase the production of senescence-associated genes (p16 and p21) and senescence-associated secreted phenotype (SASP), which includes IL-1β and IL-6. Through the JNK/p53 signal pathway, Cd exposure simultaneously accelerated AF cell senescence and promoted SASP. Following JNK inhibitor (SP600125) treatment, the expression of p53, JNK, and senescence-associated indices were all down-regulated.

**Conclusion:**

By activating the JNK/p53 signaling pathway, Cd can induce oxidative stress damage and AF cell senescence. These findings could provide a new approach for treating and preventing intervertebral disc degeneration (IVDD) caused by Cd exposure.

## Introduction

Low back pain (LBP) is a major contributor to the global disease burden and has a severe negative impact on people’s quality of life (1, 2). Over 70% of the global population experiences LBP at various stages of life (2). It is now widely accepted that one of the primary causes of LBP is intervertebral disc degeneration (IVDD) (2, 3). IVDD is a multifactorial pathological process and the leading cause of years lived in disability worldwide (4). Major molecular changes that occur in intervertebral disc degeneration (IVDD) include immune cell infiltration, extracellular matrix (ECM) breakdown, and abnormal apoptosis, senescence, and apoptosis of intervertebral disc (IVD) cells (5). Chronic tobacco smoking contributes to the amplification of senescence-inducing stresses, demonstrates inflammatory and catabolic phenotypes, deteriorates the disc microenvironment, and accelerates IVDD (6,7). In addition, Cd is one of the most important risk factors for smoking. Nevertheless, a complete explanation of the detrimental consequences of smoking on IVDD is elusive.

Over a decade is the half-life of cadmium (Cd), a toxic heavy metal that pollutes the environment. (8). Inhalation of cigarette smoke or intake of Cd-contaminated food or water is the main source of human Cd exposure (9). Cd exposure has been shown to be deleterious to humans and, causes a wide range of disorders such as cardiovascular disease, osteoporosis, and many forms of cancer (10-16). Studies have shown that Cd can affect cell function, such as apoptosis of osteogenic differentiation of bone mesenchymal stem cells (BMSCs), neuronal cells, DNA damage, apoptosis of osteoblasts, and osteoclast differentiation (17-20).

The annulus fibrosus (AF) is an important part of IVD tissue. Although there have been several studies on AF cell apoptosis, few have been on cell senescence. The senescence of IVD cells affects the functional and structural stability of IVD tissue. Organelles such as mitochondria have a significant impact on the production of reactive oxygen species (ROS) and are one of the key intracellular targets of Cd. Mitochondrial dysfunction is caused by Cd exposure, resulting in decreased ATP (adenosine triphosphate) production and massive accumulation of ROS (14).

The motivation behind this study was to investigate the impact and mechanism of Cd on AF cells and to find a feasible target for the prevention of IVDD caused by Cd exposure.

## Materials and Methods

***Isolation and culture of AF cells***

The Animal Care and Ethics Committee of Yangzhou University gave its approval to every experiment conducted for this study. From the IVD tissue of 200-260 g Sprague-Dawley (SD) rats, rat AF cells were isolated. A sodium pentobarbital overdose killed ten male SD rats. Under aseptic circumstances, the IVD tissues were extracted from the coccygeal. A dissecting microscope was then used to carefully separate the AF tissues. Then, for ten hr at 37 ^°^C, AF tissues were treated with 0.25% collagenase I. Subsequently, the tissue that had been digested was placed onto DMEM (Gibco, USA) which included 1% penicillin/streptomycin and 15% fetal bovine serum (Gibco, USA). After 5 days of culture in a 5% CO_2_ and 37 ^°^C incubator, adherent cells were AF cells after removal of tissue debris and suspended cells. The cells were passaged at a ratio of 1:3 when the cell confluence was 80-90%. Every two days, the medium was swapped out. Further tests were conducted using third-passage cells.

***Cell viability assay***

Cytotoxicity was detected by Cell Counting Kit-8 (CCK-8) according to the instructions provided by the reagent manufacturer. AF cells were seeded in 96-well plates (5,000 cells per well) and incubated overnight in a complete medium at 37 ^°^C and 5% CO_2_. The cells were treated with different concentrations of Cd at different times. Then, 10% CCK-8 was added to each well at various time points. After 1 hr of incubation, the optical density (OD) value was measured at 450 nm by a microplate reader (Bio-Rad, USA). The cell viability was assessed as follows: Cell viability (100% of control)=[(Ae-Ab)/(Ac-Ab)] x 100. Ae, Ab, and Ac represent the A450 of the treatment, blank, and control groups, respectively. Similarly, different concentrations (0-80 μM, 0-48 h) of the JNK inhibitor SP600125 (MedChemExpress, China) were cocultured with AF cells to further determine whether the JNK/p53 pathway was involved.

To further investigate whether Cd-induced senescence of AF cells was related to the JNK/p53 pathway, AF cells were pretreated with 10 μM SP600125 and 5 μM Cd for 24 hr. AF cells were then divided into the following groups: (1) control group, (2) Cd group (5 μM ClCd_2_), and (3) SP+Cd group (5 μM ClCd_2_+10 μM SP600125).

***Cell proliferation assay***

AF cells (5×10^4^ cells/well) were seeded in 12-well plates and cultured in a DMEM low-glucose medium. To evaluate cell proliferation, an EdU Cell Proliferation Kit (Beyotime, China; catalog number C0071S) was utilized. Following a 2-hour incubation period with EdU, AF cells were fixed for 15 min using 4% paraformaldehyde. The cells were then incubated for 10 min, per the manufacturer’s directions, with 0.3% Triton X-100. Cells were then incubated with ClickReactionMixture for 30 min followed by Hoechst 33342 for 10 min in the dark. Finally, the cells were observed and recorded using fluorescence microscopy and analyzed by ImageJ software (NIH, USA).

***Senescence-associated β-galactosidase staining***

A 6-well plate was seeded with AF cells at a density of 3×10^5 ^cells/well under 5% CO_2_ at 37 ^°^C. Senescence-associated β-galactosidase (SA-β-Gal) staining was carried out in accordance with the SA-β-Gal staining kit’s manufacturer’s instructions (Beyotime, China, catalog No. C0602). AF cells were examined using a fluorescent microscope, and ImageJ was used for analysis.

***RNA sequencing of the AF cell transcriptome***

TRIzol reagent was used to extract total RNA from AF cells, either with or without Cd. RNA sequencing was performed on the acquired RNA samples by Genechem Co., Ltd. in Shanghai, China. Using featureCounts software, the expression FPKM values of the associated genes in each sample were found. The DESeq technique was used to count the genes that were differentially expressed. Genes with significantly different expressions were identified as follows: ∣log2foldchange∣>1 and *P*<0.05. GO analysis was used to characterize the biological activities of differential genes, such as cellular component (CC), molecular function (MF), and biological process (BP). Using KEGG database analysis, pathways with notable enrichment of differential genes were identified (BD Company, United States).

***JC-1 assay for mitochondrial membrane potential (MMP)***

MMP was detected with a JC-1 Staining Kit (Keygen, China, catalog No. KGA603). Following a PBS wash, AF cells from various groups were incubated for 30 min in the dark with a 1.5 ml mixture of JC-1 staining solution. Following that, a cold staining buffer was used to wash the cells. MMP was then found using fluorescence microscopy (Olympus, Japan) or flow cytometry. Either ImageJ or CellQuest analysis software (BD) was used to calculate the ratio of red to green fluorescence intensity.

***ROS assay***

A ROS detection fluorescent probe-DHE kit (Keygen, China, catalog No. KGAF019) was used to quantify the amount of intracellular ROS in AF cells. AF cells were subjected to several treatments on a 12-well plate, followed by two PBS washes and an hour-long incubation at 37 ^°^C with 20 μM DHE, as per the manufacturer›s instructions. After that, ImageJ was used to analyze the cells after they were seen under a fluorescence microscope.

***Adenosine triphosphate assay***

Adenosine triphosphate (ATP) levels in AF cells were measured by an ATP Assay Kit (Beyotime, China, catalog No. S0026B). AF cells were incubated with ATP assay lysis buffer. Then, the supernatant was collected at 12,000×g for 5 min at 4 ^°^C, and the protein content was measured using a BCA Protein Assay kit (Beyotime, China, catalog No. P0010). The detection reagent was mixed with the supernatant, and a multifunction microplate reader (BioTek SynergyHTX, USA) was used for detection.

***Quantitative real-time polymerase chain reaction (qRT-PCR)***

Utilizing the TRIzol reagent (Invitrogen, USA; catalog number: 15596-026), total RNA was extracted. Following the manufacturer’s instructions, a Prime Script-RT reagent kit (Vazyme Biotech, China, catalog No. R123-01) and SYBR Premix Ex Taq (Vazyme Biotech, China, catalog No. Q111-02) were used for reverse transcription from whole RNA to complementary DNA (cDNA) and amplification of the cDNA. Using the comparative Ct technique, the expression of target genes was determined. Using Prime 5.0 software, the primers were created in accordance with the sequences found in GenBank and are presented in Table 1.

***Western blot analysis***

The BCA protein assay kit (Beyotime, China, catalog No. P0010) was used to determine the protein concentration after the whole protein was extracted from AF cells using the Whole Cell Lysis Assay (Keygen, China, catalog No. KGP250). Following sodium dodecyl sulfate-polyacrylamide gel electrophoresis, an identical protein sample from each group was placed onto a polyvinylidene fluoride membrane. After that, the membranes were blocked with 5% nonfat milk for 2 hr at room temperature and then incubated with primary antibodies against GAPDH (Proteintech, China, catalog No. 20536-1-AP)(1:3000), p16 (Proteintech, China, catalog No. 10883-1-AP)(1:1000), p21 (Proteintech, China, catalog No. 10355-1-AP)(1:1000), p53 (Proteintech, China, catalog No. 10442-1-AP), JNK (Proteintech, China, catalog No. 10589-1-AP), p-JNK (Proteintech, China, catalog No. 10986-1-AP), collagen I (Proteintech, China, catalog No. 20366-1-AP), IL-1β (Proteintech, China, catalog No. 20510-1-AP), and IL-6 (Proteintech, China, catalog No. 20314-1-AP) overnight at 4 ^°^C. The membranes were incubated with secondary antibodies (Proteintech, China, catalog No. SA00001-2)(1:5000) for two hours on a shaker at room temperature after being washed three times with Tris-buffered saline and 0.1% Tween 20 (TBST). Next, an improved chemiluminescence method was used to view the membranes, and ImageJ was used to analyze the relative protein content.

***Immunofluorescence staining***

AF cells were seeded in a 12-well plate, treated with 0, 5 μM/l Cd, and 5 μM/l Cd+10 μM/l SP600125, and then prepared for immunofluorescence staining. Before fixation in 4% paraformaldehyde for 30 min, the samples were washed with PBS three times. Afterward, the samples were washed with PBS an additional three times and treated with 0.5% Triton X-100 for 20 min. Then, the samples were blocked for 30 min in 5% goat serum albumin and incubated with anti-p-JNK, p53, p21, p16, and collagen I antibodies (10883-1-AP, 10355-1-AP, 20556-1-AP, 10986-1-AP, 20366-1-AP, respectively; Proteintech) overnight at 4 ^°^C. Following PBST washing, the samples were incubated for one hour in the dark with a secondary antibody conjugated with a fluorophore. Following that, the samples were counterstained for five min in the dark using 4′,6-diamidino-2-phenylindole. A fluorescence microscope captured images of the sections, which were then processed using ImageJ software.

***Cell cycle assay***

Serum-free media was used to seed AF cells in 6-well plates during an overnight period. The cells were then gathered and preserved for the night in 75% ethanol. After 30 min of exposure to RNase A (Keygen, China, catalog number KGA511), the cells were examined using flow cytometry (BD Company, USA) to determine the phases of the cell cycle.

***Statistical analysis***

The Statistical Package for the Social Sciences (SPSS) software (version 26; IBM, Chicago, Illinois) was used to analyze all of the data. The mean±SD is used to express the quantitative data. The one-way ANOVA method was used to analyze the data from several separate groups. To examine the variations between the two groups, the student’s t-test was applied (*P*<0.05).

## Results

***Cd-induced senescence of AF cells***

**Figure 1:**
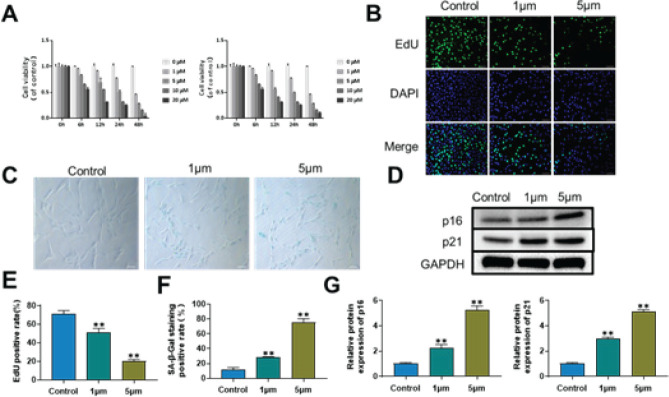
Toxicity effect of cadmium (Cd) on annulus fibrosus cells (AF cells)

The CCK-8 assay was used to analyze how Cd affected the viability of AF cells. Higher concentrations of Cd (>10 μM) exerted an essential inhibitory effect on cell viability, but Cd (0-5 μM) showed an appropriate inhibiting effect on cell viability for 12 hr, as shown in Figure 1a. Consequently, Cd concentrations of 0, 1, and 5 μM were used for the next experiment, and 12 hr was chosen as the intervention time. The ability of AF cells to proliferate steadily declined as Cd concentrations increased, as seen in Figure 1b. The SA-β-Gal staining results demonstrated that AF cell senescence rose as Cd concentrations increased (Figure 1c). Following that, the western blotting outcomes demonstrated that as Cd concentration increased, so did the expression levels of p16 and p21 (Figure 1d). These findings demonstrated that Cd could induce AF cell senescence.

***Analysis of Transcriptome Sequencing after Cd Treatment***

**Figure 2:**
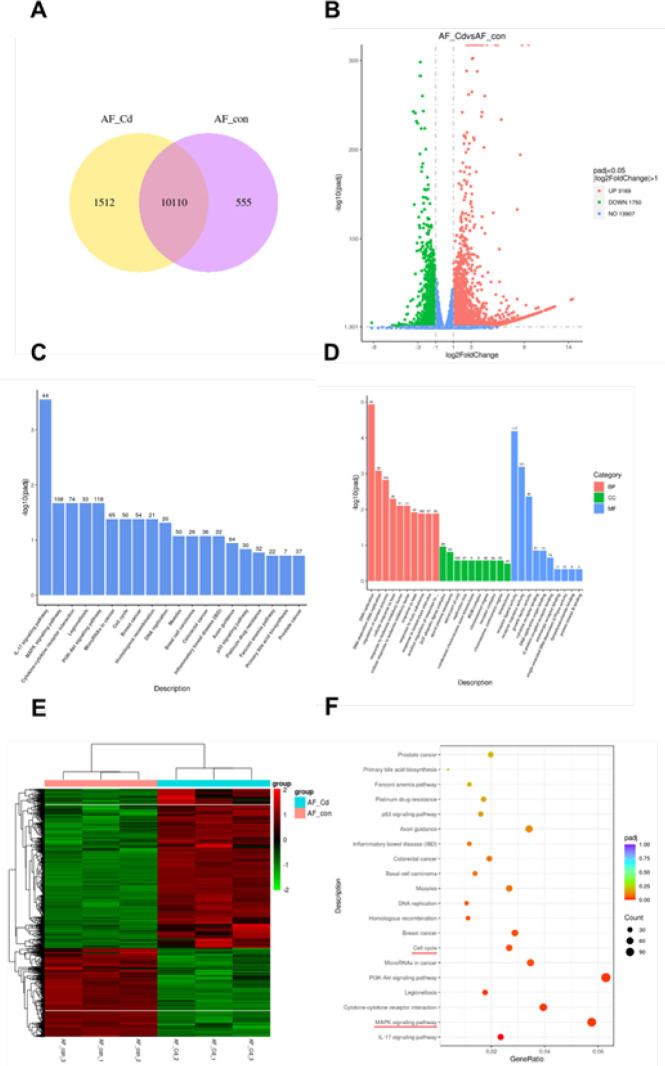
Bioinformatics analysis based on mRNA transcriptome sequencing results after treatment with cadmium (Cd)

Venn diagram of coexpression showing 1512 genes that were uniquely expressed in Cd-treated AF cells (Figure 2a). The volcano plot showed that the significantly different gene JNK was up-regulated after Cd treatment (Figure 2b, f). Among the differentially expressed genes in the transcriptome, the key signaling molecule JNK in the MAPK signaling pathway was significantly up-regulated. Among the 15 KEGG pathways with significant differences in enrichment analysis, the p53, MAPK, and cell cycle signaling pathways were found (Figure 2c, f). The findings suggested that Cd-induced senescence of AF cells involved the JNK/p53 signaling pathway.

***Effects of the JNK/p53 signaling pathway on Cd-induced AF cell senescence***

**Figure 3:**
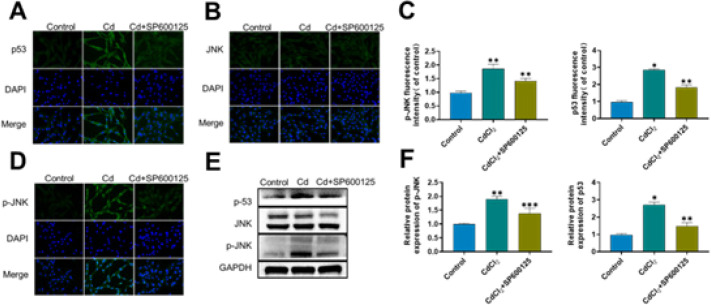
Cadmium (Cd) activates the JNK/p53 signaling pathway

According to the CCK-8 results, 5 μM Cd and 10 μM SP600125 were used as the final treatment concentrations (Figure 1a). We utilized the JNK inhibitor SP600125 to examine how the JNK/p53 signaling pathway affected the senescence of AF cells induced by Cd. As demonstrated in Figure 3a, Cd markedly raised the expression of JNK phosphorylation to JNK phosphorylation and the expression of p53. On one hand, following SP600125 treatment, the expression levels of p-JNK and p53 were down-regulated (Figure 3a, d). In addition, these results were similarly confirmed by immunofluorescence. The Cd group showed considerably higher p-JNK and p53 expression levels compared to the control group, but SP600125 treatment resulted in significantly lower p-JNK and p53 expression levels.

The JNK/p53 pathway can be activated by Cd, according to the results above (Figure 3e).

**Figure 4:**
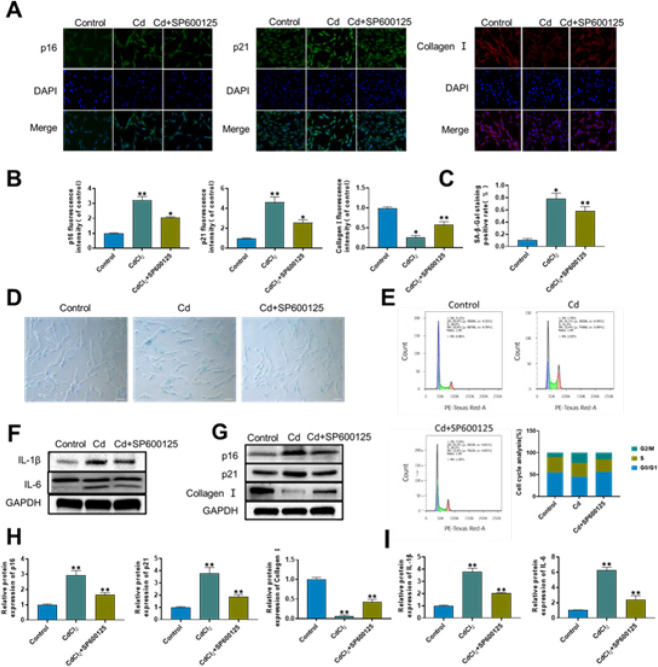
Cadmium (Cd) induces senescence of annulus fibrosus cells by activating the JNK/p53 signaling pathway

To determine the intracellular fluorescence, proteins, and mRNA expression of p16 and p21, immunofluorescence and western blot analysis were utilized (Figure 4a, g). The findings demonstrated that there was a clear up-regulation of p16 and p21 expression in the Cd group when compared to the control group. When comparing the SP+Cd group to the Cd group, the fluorescence intensity and protein expression levels of p16 and p21 were, however, down-regulated (Figure 4a, g). Results of the qRT-PCR also demonstrated that Cd group’s mRNA expression levels of p16, p21, and p53 were significantly higher than those of the control group. However, in the SP+Cd group, pretreatment with SP600125 substantially mitigated this impact. Figure 4e illustrates that in comparison to the control group, a greater proportion of AF cells in the Cd group exhibited cell cycle arrest in the G2/M phase. Following pretreatment with SP600125, the proportion of AF cells arrested in the G2/M phase dropped, revealing that SP600125 could mitigate Cd-induced cell cycle arrest. The Cd group had considerably increased levels of IL-6 and IL-1β expression compared to the control group, as depicted in Figure 4f. Nevertheless, with SP600125 treatment, IL-6 and IL-1β expression levels were lower than those of the Cd group, suggesting that SP600125 may be able to prevent the upward trend in IL-6 and IL-1β. Under SP600125 treatments, type I collagen gene expression was dramatically down-regulated compared to the control group, but type I collagen expression increased significantly in the Cd group (Figure 4a). Western blot testing was utilized as well to confirm these findings. Western blot investigation revealed that Cd group’s type I collagen expression level was lower than that of the control group, as seen in Figure 4g. As compared to the Cd group, the SP+Cd group showed higher type I collagen expression levels.

These findings showed that Cd might promote AF cell senescence by activating the JNK/p53 signaling pathway. Furthermore, inhibition of JNK/p53 signaling attenuated Cd-induced cell senescence.

***Function of mitochondria in senescence of Cd-induced AF cells***

**Figure 5:**
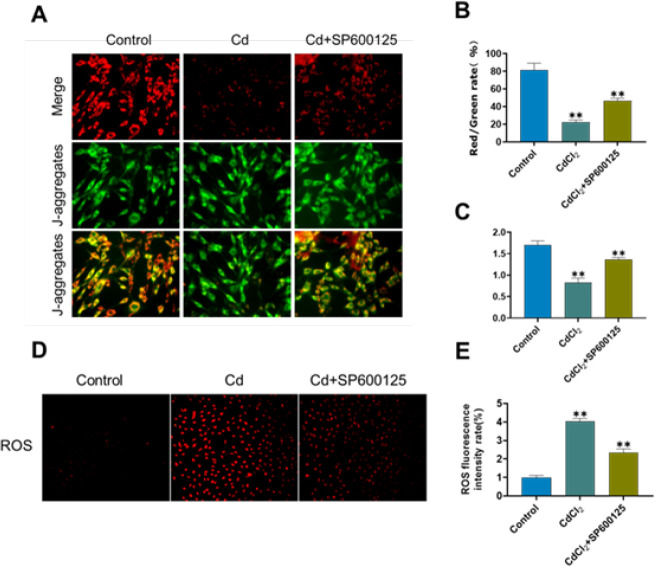
Mitochondrial dysfunction in cadmium-induced annulus fibrosus cell senescence

To determine the MMP level, a mitochondrial membrane potential assay kit (JC-1) was utilized. When compared to the control group, the MMP level was significantly decreased in the Cd group; however, upon pretreatment with SP600125, the MMP level increased (Figure 5a). To further observe the function of mitochondria, intracellular ATP levels were measured by an ATP assay kit. The findings demonstrated that following pretreatment with SP600125, the ATP level was higher in the Cd group than in the control group, and that the ATP level in the Cd group had dramatically dropped (Figure 5c). ROS fluorescence intensity in the Cd group was considerably greater than in the control group, as Figure 5d illustrates. However, the ROS level diminished following pretreatment with SP600125, showing the inhibitors can reduce the ROS level. Therefore, Cd can promote AF cell senescence through oxidative stress damage to mitochondria.

## Discussion

Since AF is an important part of IVD tissue, the structural stability of AF is crucial to preventing IVDD. The mechanism underlying IVDD is still unclear, though. One common environmental pollutant that can lead to a severe bone metabolism disorder is Cd (21). All living things are seriously threatened by this type of environmental contamination of the water, soil, air, and food (22). Additionally, a variety of studies found that Cd has a negative effect on human wellness and is a factor in several illnesses, including IVDD, osteoporosis, cardiovascular disease, and cerebrovascular disease (23-28). However, the mechanism behind these processes is not fully clear.

One of the primary organs targeted by Cd damage is the bone, which can result in conditions associated with the bones, including osteoporosis and osteoarthritis. According to certain research, individuals with spinal osteoarthritis had blood concentrations of Cd that were much higher than those of the control group, which indicates that Cd toxicity is closely related to spinal disease (29). In addition, Cd can induce apoptosis of AF cells in intervertebral discs, thus promoting IVDD, and the cytotoxicity of Cd to intervertebral discs is mainly due to mitochondrial dysfunction mediated by oxidative stress (28). Therefore, Cd exposure may accelerate the process of IVDD. Cd exposure accelerates cellular senescence and leads to aging, increasing the risk of osteoarthritis mediated by biological aging (30). Many investigations have been conducted on the kidneys’ aging process. Given the available information, senior people who have been exposed to Cd have higher incidence and severity of renal illness (31). Studies have reported that Cd can significantly induce bone marrow-derived mesenchymal stromal cell senescence through activation of the NF-κB signaling pathway (32). Utilizing SA-β-Gal labeling and western blot examinations, various concentrations of Cd were employed to verify the function of Cd in AF cells. We discovered that when Cd concentrations increased, accompanied by the percentage of senescent cells and the up-regulation of p16 and p21 protein expression. Therefore, Cd can lead to senescence of AF cells.

Excessive intracellular or extracellular stress or damage results in senescence, an irreversible type of prolonged cell cycle arrest (33). Mitochondrial dysfunction and DNA damage result from Cd-induced cellular damage and oxidative stress (21). Excessive formation of ROS spurred on by oxidative stress can result in damage to proteins, DNA, and mitochondria (34, 35). In addition, one of the main causes of cell senescence is excessive accumulation of ROS inside the cell (34, 36, 37). The content of ROS in AF cells also increased significantly after Cd treatment in the present study. Thus, the pathways of Cd-induced AF cell senescence could involve mitochondrial dysfunction and ROS accumulation. The reduction in ROS content observed following SP600125 treatments is consistent with the involvement of the JNK/p53 signaling pathway in oxidative stress-induced damage to AF cells, as per our findings.

Interestingly, mitochondria seem to be the main target of Cd poisoning (38). The regulation of cellular senescence depends heavily on mitochondria, the center for metabolism and signal transduction (39, 40). MMP levels decrease and the quantity of mitochondria increases all over the procedure of cell senescence. Production of ROS and the release of enzymes from the mitochondria may result from a decline in mitochondrial activity (41). Accordingly, mitochondrial homeostasis has a major impact on senescence. We evaluated whether mitochondria contributed to cell senescence to find out more about the mechanism underlying Cd-induced cell senescence. The findings indicated that the J-aggregates’ content in matrix metalloproteinases and the amount of ATP decreased significantly after Cd treatment, while the J-aggregates’ content and the amount of ATP partially recovered after SP600125 treatment. Therefore, lowering the production of ROS and modulating the function of mitochondria may help reverse Cd-induced AF cell senescence.

Numerous studies have attempted to explain the Cd-activated MAPK signaling pathway, including p38, ERK, and JNK (42-45). Activation of the JNK signaling pathway may accelerate cellular senescence (46). There is growing evidence to suggest that dysregulation of JNK has a connection with biological processes that contribute to aging, such as irreversible senescence (47). We found that Cd can activate the phosphorylation of JNK. To validate the role of JNK in cellular senescence, AF cells were pretreated with SP600125 before treatment with Cd. The results indicated that SP600125 reversed the accumulation of p-JNK caused by Cd. The JNK/p53 pathway anticipates the regulation of SASP and p53. In the meantime, JNK and p53’s interaction may accelerate the development of SASP (48). L-carnitine reduced aging-related adipose tissue dysfunction and suppressed SASP via the JNK/p53 pathway (49). The findings of this investigation showed that Cd may improve the expression levels of IL-1β and IL-6, and that pretreatment with SP600125 reversed the consequences of the study. Accordingly, our results revealed that the JNK/p53 signaling pathway is a potential mechanism of Cd-induced AF cell senescence. Cd can cause AF cell senescence and further reduce the stability of AF tissue, increasing the risk of rupture, which may eventually lead to IVDD. The senescence of AF cells induced by Cd is involved in the occurrence of IVDD, which may mediate the consequences of exposure to Cd on IVDD. These findings indicated that activation of the JNK/p53 signaling pathways is consistent with our transcriptome sequencing results. More importantly, it may be feasible to prevent IVDD in cases of Cd exposure by targeting the JNK/p53 signaling pathway. In summary, the senescence of AF cells caused by Cd has been connected with the JNK/p53 signaling pathway.

However, there are several limitations in this study. Recent evidence found that Cd can regulate cell biological processes through multiple signaling pathways, including nuclear factor erythroid 2-related factor 2 and Kelch-like ECH-associated protein 1 (Nrf2- Keap1)(50), miR-34a/sirtuin-1/p53 (51), and extracellular signal-regulated protein kinase (ERK)(52), as also illustrated by transcriptome sequencing analysis. It is challenging to determine if JNK/p53 is the only signaling mechanism that regulates Cd-induced AF cell senescence because we have not examined other signaling pathways implicated in this process. Therefore, more research is required to determine the underlying mechanism of Cd-induced senescence in AF cells.

**Table 1:** Sequences of primers used for real-time polymerase chain reaction

Gene	Primer/Probe Sequence
GAPDH	Forward 5'-CTGGAGAAACCTGCCAAGTATG-3' Reverse 5'-GGTGGAAGAATGGGAGTTGCT-3'
p16	Forward 5'-CCGATACAGGTGATGATGATGG-3' Reverse 5'-CGGAGGAGAGTAGATACCGCAAA-3'
p21	Forward 5'-AGTTGGAGCTGGTGGCGTAG-3' Reverse 5'-AATACACAAAGAAAGCCCTCCC-3'
p53	Forward 5'-ATGGAGGATTCACAGTCGGATAT-3' Reverse 5'CGCTGTGGTGGGCAGAATAT-3'

A: Cell counting kit-8 (CCK-8) results of annulus fibrosus cells (AF cells) treated with different concentrations and times of Cd and SP600125; B: EdU assay results of AF cells treated with different concentrations of Cd. Green fluorescence represents proliferating cells, and blue fluorescence represents the cell nucleus. (scale bar=50 μm); C: Senescence-associated β-galactosidase (SA-β-Gal) staining results of AF cells in different groups. Senescent cells exhibit blue-stained high expression of SA-β-Gal. (scale bar=50 μm); D: Expression of senescence-related proteins (p16 and p21) cultured with different concentrations of Cd; E: Quantitative analysis of EdU results; F: Quantitative analysis of SA-β-Gal staining results; G: Quantitative analysis of p16 and p21 protein expression results

All data are expressed as the mean±SD. **P<0.01 compared with the control group

A: Coexpression Venn diagram; B: Volcano plots of differentially expressed genes in the transcriptome; C: Pathway analysis from the annulus fibrosus cell (AF cell) transcriptome; D: Gene Ontology (GO) analysis of the transcriptome of AF cells treated with Cd. E: Differential expression gene clustering heatmap; F: Bubble plot of the Kyoto Encyclopedia of Genes and Genomes (KEGG) enrichment analysis results

A-B: Immunofluorescence staining results of p53 and JNK in annulus fibrosus cells (AF cells) after Cd treatment. (scale bar=50 μm); C: Quantitative analysis of the fluorescence expression levels of JNK and p53; D: Immunofluorescence staining results of p-JNK in AF cells after Cd treatment. (scale bar=50 μm); E: Expression levels of p53, JNK, and p-JNK protein in different groups; F: Quantitative analysis of p-JNK and p53 protein expression results

All data are expressed as the mean±SD. *P<0.05,**P<0.01 and ***P<0.001 compared with the control group

A: Immunofluorescence staining results of p16, p21, and Collagen I in annulus fibrosus cells (AF cells) after Cd treatment. (scale bar=50 μm); B: Quantitative analysis of the fluorescence expression levels of p16, p21, and Collagen I; C: Quantitative analysis of SA-β-Gal staining results; D: Senescence-associated β-galactosidase (SA-β-Gal) staining results of AF cells in different groups. Senescent cells exhibit blue-stained high expression of SA-β-Gal. (scale bar=50 μm); E: Cell cycle results of AF cells in different groups; F: Expression of IL-1β and IL-6 protein in different groups; G: Expression of p16, p21, and Collagen I protein in different groups; H: Quantitative analysis of p16, p21, and Collagen I protein expression results; I: Quantitative analysis of IL-1β and IL-6 protein expression results

All data are expressed as the mean±SD. *P<0.05 and **P<0.01 compared with the control group

A: Results of mitochondrial membrane potential (MMP) in different groups detected by fluorescence. Red fluorescence represents the mitochondrial aggregate JC-1, and green fluorescence indicates the monomeric JC-1. (scale bar=50 μm); B: Quantitative analysis of MMP results; C: ATP levels of AF cells treated with Cd in different groups; D: Results of reactive oxygen species (ROS) in different groups detected by fluorescence. Red fluorescence represents a high level of ROS (scale bar=50 μm); E: Quantitative analysis of ROS results

All data are expressed as the mean±SD. **P<0.01 compared with the control group

## Conclusion

For the first time, the mechanism of Cd-induced AF cell senescence was assessed in this study. It was concluded that Cd exposure can induce senescence and mitochondrial dysfunction in AF cells. The JNK/p53 signaling pathway could play an essential part in the senescence of AF cells triggered by Cd *in vitro*. These findings present a viable foundation for IVDD reconstruction of Cd toxicity treatments.

## Data Availability

All data of the study are available from the corresponding author upon request. 

## Authors’ Contributions

L X and Zh WJ contributed to data curation, Writing-Original draft preparation,Investigation,conceptualization and methodology, contributed equally to this work; H M and Zh Y contributed to Visualization, Validation; W JC and Zh L performed supervision, writing- reviewing, editing and share corresponding author.

## Conflicts of Interest

The authors declare no conflicts of interest regarding the publication of this paper, and the manuscript is approved by all authors for publication.
